# Evaluation of the Efficacy of a Serum Containing Niacinamide, Tranexamic Acid, Vitamin C, and Hydroxy Acid Compared to 4% Hydroquinone in the Management of Melasma

**DOI:** 10.1111/jocd.70097

**Published:** 2025-03-10

**Authors:** Juliane Rocio, Jean Christophe Pittet, Mukta Sachdev, Natalia Kovylkina, Claire Deloche Bensmaine, Thierry Passeron

**Affiliations:** ^1^ Institute of Dermatology & Aesthetics of Rio de Janeiro Rio de Janeiro Brazil; ^2^ Orion Concept Tours France; ^3^ MS Skin Centre and MSCR Bangalore India; ^4^ Vichy Laboratoires International France; ^5^ Department of Dermatology, CHU Nice Université Côte d'Azur Nice France; ^6^ C3M, INSERM U1065 Université Côte d'Azur Nice France

**Keywords:** depigmentation, dermocosmetic, hydroquinone, melasma

## Abstract

**Introduction:**

Melasma is a common skin condition that remains challenging to treat. Hydroquinone at 4% (HQ4%) is a frequently prescribed depigmenting compound that has been associated with potential side effects.

**Objective:**

This study assessed the benefit in melasma of an anti‐hyperpigmentation serum (Serum B3 containing 5% niacinamide, 1% tranexamic acid, 0.2% of a stabilized form of vitamin C, and different hydroxy acids) compared to HQ4%.

**Materials and Methods:**

In a single‐site, investigator‐blind, randomized study, 60 females aged between 20 and 50 years with facial melasma received Serum B3 for 5 months (Group 1) or HQ4% for 3 months followed by Serum B3 for an additional2 months (Group 2). Endpoints were Melasma Area and Severity Index (MASI), modified MASI (mMASI), Investigational Global Assessment, erythema, clinical cutaneous parameters, and safety. Subjects assessed quality of life (QoL) and cosmetic acceptability. Confocal reflecting microscopy was performed.

**Results:**

A significant (*p* < 0.001) reduction in pigmentation was seen in both groups after 3 months. A higher erythema score was noted in Group 2. Hydration and skin barrier function performed better in Group 1. QoL significantly (*p* < 0.001) improved in both groups after 84 days. Serum B3 was tolerated better than HQ, and subjects appreciated Serum B3. Melanin density reduction was similar for both groups after 3 and 5 months.

**Conclusion:**

Serum B3 used for 5 months and HQ4% applied for 3 months, followed by a 2‐month use of Serum B3, had a similar efficacy profile, with Serum B3 having a better local tolerance and patient acceptability.

## 1 Introduction

Melasma is a common skin hyperpigmentation condition that affects the skin. It presents with brown macules and patches which are symmetrically disposed [1, 2]. It is more commonly seen in women between 20 and 50 years of age, of Fitzpatrick's phototypes III–V, and among multiple and mixed ethnicities. Multiple factors are involved in its etiopathogenesis which have become better known over recent years and include genetic predisposition, sunlight exposure, and hormonal factors [1, 3, 4]. Increasing data suggest that melasma is a photo‐aged disorder affecting predisposed individuals [2]. Skin aging is clinically characterized by pigmentation, atrophy, a loss of elasticity, and an impaired recovery response against damage, resulting in subsequent pathologic skin disorders. Skin aging is triggered by intrinsic and extrinsic pathways. Genetic and hormonal factors are inherently associated with unavoidable skin aging, while environmental factors such as ultraviolet radiation are extrinsically associated with preventable skin aging. Recently, cellular senescence has been suggested to be a key player in skin aging hyperpigmentation. Moreover, various other skin cell types, such as keratinocytes, melanocytes, fibroblasts, and endothelial cells, play a role in skin aging. Their cross talk during the aging process may play an important role in melanogenesis and the subsequent aging‐related pigmentation [5, 6].

Melasma is a chronic disorder with almost constant relapses that require maintenance treatment and photoprotection throughout the year. It has a well‐demonstrated, deleterious impact on a patient's quality of life [7, 8].

Currently, several topical agents are proposed to manage melasma [9, 10]. One range of products induces a disruption of the melanogenesis process that leads to pigment production within the melanocytes. Of these products, hydroquinone (HQ) alone at 4% or in combination is considered the leading topical agent to manage melasma, due to its superior depigmenting efficacy [11, 12, 13, 14, 15, 16]. HQ inhibits melanin synthesis. However, several side effects such as ochronosis, erythema, scaling, irritation, and burning sensation are reported [12, 13, 17]. Another approach is improving skin turnover using agents such as glycolic acid, lactic acid, retinoic acid, and linoleic acid (topical treatment and chemical peels) [18, 19].

A novel dermocosmetic serum (Liftactiv B3; Laboratoire Vichy International, France, hereafter Serum B3) containing 5% niacinamide, 1% tranexamic acid (TA), 0.2% CG (C‐Glucoside, a stabilized form of vitamin C), and a cocktail of different hydroxy acids, including 1.5% glycolic acid, has been developed to manage melasma. Niacinamide is the biologically active form of niacin (vitamin B3) and reduces skin pigmentation by decreasing the accumulation of melanin [20, 21]. Topical niacinamide may be considered a beneficial and safe therapeutic choice for the management of melasma [22, 23, 24, 25]. TA has a hypo‐pigmenting effect on melasma lesions [15, 26, 27, 28, 29, 30]. Vitamin C is one of the most commonly used topical products to manage melasma, and it is usually associated with other depigmenting ingredients [23]. Previous investigations have shown that a topical combination of TA 2% and vitamin C 2% offers an effective tool for treating resistant melasma [27]. Alpha hydroxy acids (AHAs) include glycolic, citric, and lactic acid that can be used as exfoliating agents to remove dead skin cells and stimulate the growth of smooth and evenly pigmented new skin [31]. AHA peel improves epidermal melasma [31, 32]. Glycolic acid (GA) is used in the treatment of melasma either as a sole agent or in combination with other agents [30, 33, 34]. It has depigmenting effects through the loss of cell–cell cohesion, desquamation, and inhibition of melanogenesis. It was hypothesized that a fixed combination of these compounds could potentiate their individual benefit, combined with better local tolerance compared to hydroquinone.

This study compared the efficacy of Serum B3 and hydroquinone at 4% (HQ4%, hydroquinone 40 mg/g, Germed, Brazil) in adult women with melasma.

## 2 Materials and Methods

### 2.1 Settings

This was a single‐center, investigator‐blind, cosmetic study which received ethics committee approval (IEC Rio de Janeiro, Brazil, CEP: 22.270‐005, No.: 5.418.666, May 2022) and respected the principles of the Declaration of Helsinki and Good Clinical Practices. All subjects provided written informed consent prior to inclusion in the study.

### 2.2 Subjects

Sixty women from different ethnicities, aged between 20 and 50 years, with phototypes II–VI, with a clinically confirmed symmetrical epidermal melasma on each side of the face (right and left) and with at least a 1‐year evolution of melasma, were recruited for this study. Moreover, subjects had to have self‐declared sensitive skin. Sensitive skin was defined according to the definition given by Misery et al. in 2017 [35].

### 2.3 Products

Subjects were randomized into either Group 1 (Serum B3) or Group 2 (HQ4%) after a two‐week washout phase. During this washout, all subjects applied a facial moisturizer (Mineral 89 booster − Hyaluronic Acid + Vichy Spring Water, Vichy) in the morning and a facial sunscreen (SPF 50+ UVA PF 46) twice a day. At baseline, subjects in Group 1 applied Serum B3 for 5 months twice daily. Subjects in Group 2 applied HQ4% once daily in the evening for 3 months as previously tested by Lima et al. in 2021 [36]. After these first 3 months, subjects in Group 2 received Serum B3 twice daily for a further 2 months. All subjects applied sunscreen twice daily during the entire study.

### 2.4 Assessments

At baseline and at all subsequent visits (after 1, 2, 3, 4 and 5 months), the investigator evaluated the evolution of melasma using the Melasma Area and Severity Index (MASI, scoring from 0 = no melasma to 48 points = very severe melasma, the primary endpoint) and its modified version (mMASI), made a global assessment (IGA) of erythema and hyperpigmentation, assessed fine lines, skin tone, radiance, skin texture, and skin elasticity on a scale from 0 to 5, as well as any possible adverse events and local intolerance [37, 38]. Global efficacy was assessed using a 5‐point scale (0 = not efficacious to 4 = very efficacious); local tolerance on the treated area was evaluated using a 5‐point skin reaction scale (0 = not reactive to 4 = very reactive).

Subjects answered a cosmetic acceptability questionnaire at the end of the comparative phase and completed a melasma quality of life (QoL) questionnaire (MelasQoL) at the selection visit (14 days prior to baseline visit) and after 3 and 5 months [39].

Instrumental measurements included chromametry (L*, a*, b* and individual typology angle (ITA) values) on a defined melasma area (Chromameter CR400, Minolta Japan), and skin hydration (Corneometer, CM 825, Courage + Khazaka, Germany), as well as transepidermal water loss (TEWL; Tewameter, Courage + Khazaka, Germany). The used corneometer obtains exact and reproducible values of the hydration level of the skin surface. The measurement is based on capacitance measurement of a dielectric medium. The device measures the change in the dielectric constant due to skin surface hydration by capacitance differences of a precision capacitor.

In total, 23 subjects underwent Reflecting Confocal Microscopy (RCM) (VivaScope 3000, VivaScope, Germany) [40]. RCM evaluated the product effect on the epidermis, dermoepidermal junction, and superficial dermis at baseline and after 3 and 5 months of product use [41].

### 2.5 Statistical Analysis

Quantitative variables were summarized using means, medians, and standard deviation (SD). Qualitative variables were summarized as counts and percentages. For each parameter and treatment group, a graphical representation of the means ± 95% CI was produced to visually assess the evolution across time. The percentage change at time point d (after baseline, Month 0) was calculated on the mean value observed for each parameter (where applicable) and treatment group. For instrumental measurements, MASI, mMASI Score, and MelasQOL, Student's *t*‐test for paired samples and the Wilcoxon signed rank test were used. For the IGA assessment and clinical scores, the Wilcoxon signed rank test and Mann–Whitney *U* test were applied. For global efficacy and global tolerance, the one‐sample Wilcoxon signed rank test and Mann–Whitney *U* test were used. The null hypothesis was rejected if a *p* value < 0.05 (5% significance level) was produced by the statistical procedure. Microsoft Excel 2010 and IBM SPSS version 19.0 were used for the statistical analysis.

## 3 Results

Detailed demographic data at baseline are provided in Table 1. Baseline melasma data are given in Table 2, Table 3 details skin quality, and Table 4 details chromametry baseline data.

**TABLE 1:** Demographic data at baseline.

		Serum B3	Hydroquinone 4%	Total
Age (years)	Mean ± SD	42.9 ± 4.1	42.3 ± 5.5	42.6 ± 4.8
	Min; Max	37.0; 50.0	29.0; 50.0	29.0; 50.0
	Median	43.0	41.5	42.0
Ethnicity (*n*; %)	African American/Black	5 (15.2%)	4 (12.5%)	9 (13.8%)
	Latin/Hispanic (non‐Caucasian)	4 (12.1%)	6 (18.8%)	10 (15.4%)
	Asian	0 (0.0%)	0 (0.0%)	0 (0.0%)
	Caucasian	4 (12.1%)	4 (12.5%)	8 (12.3%)
	Other (multiple ethnicities)	20 (60.6%)	18 (56.3%)	38 (58.5%)
Phototype (*n*; %)	I	0 (0.0%)	0 (0.0%)	0 (0.0%)
	II	3 (9.1%)	3 (9.4%)	6 (9.2%)
	III	9 (27.3%)	6 (18.8%)	15 (23.1%)
	IV	14 (42.4%)	12 (37.5%)	26 (40.0%)
	V	5 (15.2%)	11 (34.4%)	16 (24.6%)
	VI	2 (6.1%)	0 (0.0%)	2 (3.1%)
Sensitive skin (*n*; %)	No	14 (42.4%)	12 (37.5%)	26 (40.0%)
	Yes	19 (57.6%)	20 (62.5%)	39 (60.0%)

Abbreviations: *n*, number; SD, standard deviation.

**TABLE 2:** Melasma data at baseline.

		Serum B3	Hydroquinone 4%
Symmetrical melasma (*n*; %)	No	0 (0.0%)	0 (0.0%)
	Yes	33 (100.0%)	32 (100.0%)
MASI score	Mean ± SD	14.93 *±* 6.96	12.46 *±* 5.15
	Min; Max	5.2; 31.4	3.9; 23.0
	Median	15.60	12.30
mMASI score	Mean ± SD	8.21 *±* 3.88	6.83 *±* 2.79
	Min; Max	2.8; 17.5	2.0; 13.2
	Median	8.50	6.45
	Mean ± SD	0	0
IGA hyperpigmentation	Mean ± SD	3.4 *±* 0.8	3.3 *±* 0.8
	Min; Max	2; 5	1; 4
	Median	4.0	3.5
IGA erythema	N	32	32
	Mean ± SD	1.4 *±* 1.0	1.2 *±* 1.0
	Min; Max	0; 3	0; 3
	Median	1.0	1.0

Abbreviations: IGA, Investigator Global Assessment; MASI, Melasma Area and Severity Index; mMASI, modified MASI; *n*/*N*, number; SD, standard deviation.

**TABLE 3:** Skin quality and instrumental data at baseline.

		Serum B3	Hydroquinone 4%
	Median	4.0	3.5
L*	Mean ± SD	53.27 ± 4.94	51.93 ± 5.29
	Min; Max	39.2; 60.4	39.6; 60.7
	Median	55.18	53.40
a*	Mean ± SD	10.64 ± 1.42	10.88 ± 1.36
	Min; Max	6.9; 14.2	8.0; 13.2
	Median	10.68	10.89
b*	Mean ± SD	17.27 ± 1.62	17.53 ± 2.10
	Min; Max	12.4; 20.2	13.2; 22.3
	Median	17.47	17.81
ITA	Mean ± SD	10.03 ± 16.28	5.90 ± 17.66
	Min; Max	−41.1; 33.0	−36.6; 32.6
	Median	16.33	10.67
TEWL (g/m^2^/h)	Mean ± SD	15.73 ± 2.30	16.86 ± 3.22
	Min; Max	10.4; 20.3	11.9; 24.3
	Median	15.79	16.28

Abbreviations: ITA, individual typology angle; TEWL, transepidermal water loss.

**TABLE 4:** Chromametry data at baseline.

		Serum B3	Hydroquinone 4%
L*	Mean (±SD)	53.27 (4.94)	51.93 (5.29)
	Min; Max	39.2; 60.4	39.6; 60.7
	Median	55.18	53.40
a*	Mean (±SD)	10.64 (1.42)	10.88 (1.36)
	Min; Max	6.9; 14.2	8.0; 13.2
	Median	10.68	10.89
b*	Mean (±SD)	17.27 (1.62)	17.53 (2.10)
	Min; Max	12.4; 20.2	13.2; 22.3
	Median	17.47	17.81
ITA	Mean (±SD)	10.03 (16.28)	5.90 (17.66)
	Min; Max	−41.1; 33.0	−36.6; 32.6
	Median	16.33	10.67

Abbreviation: ITA, individual typology angle.

All subjects were women; the mean age was 42.9 ± 4.1 years. All had had symmetrical melasma for at least 1 year prior to inclusion. No significant group difference was observed for any of the parameters assessed at baseline.

**FIGURE 1:**
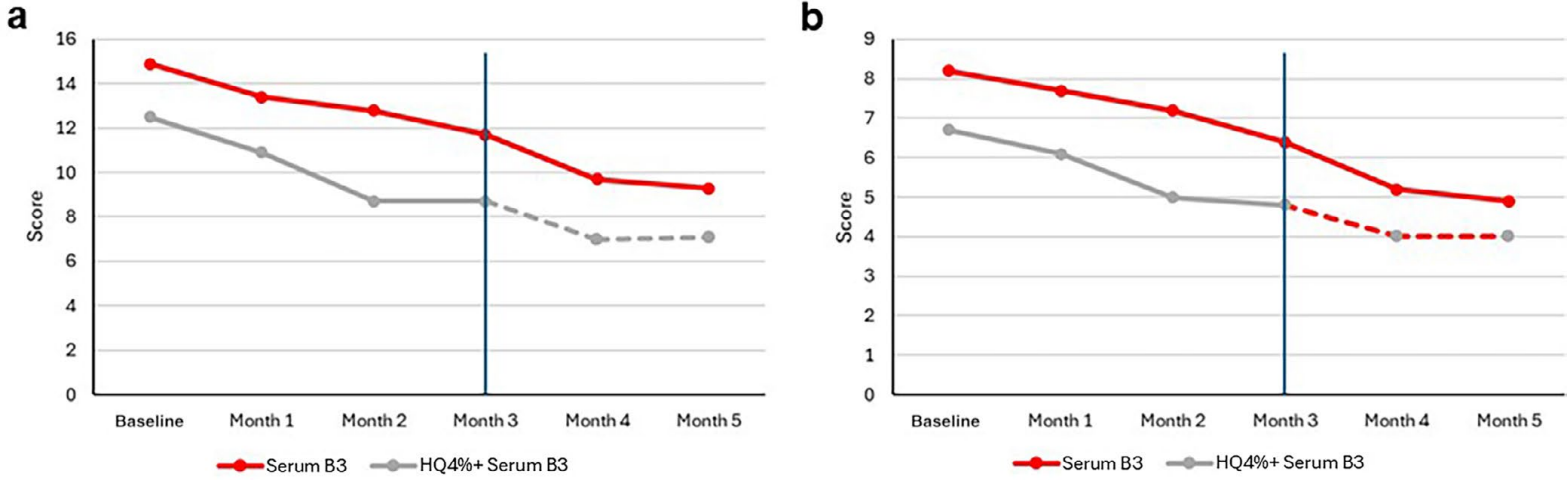
MASI and mMASI over time. (A) MASI. (B) mMASI. Both the MASI and mMASI showed a progressive decrease in both groups, with no significant between‐group difference after 3 months of product use. This trend was maintained during Months 4 and 5.

A progressive decrease of the MASI score was observed in both groups with no significant between‐group difference after 3 months of product use (Group1: −3.5 ± 2.7 points; Group 2: −3.7 ± 2.9 points). This trend was maintained during Months 4 and 5 (Group 1: Month 4: −4.7 ± 2.3, Month 5: −5.7 ± 3.2; Group 2: Month 4: −5.5 ± 3.3, Month 5: −5.4 ± 3.2); mMASI paralleled these scores. Figure 1A shows the evolution of the MASI score over time and Figure 1B that of the mMASI.

**FIGURE 2:**
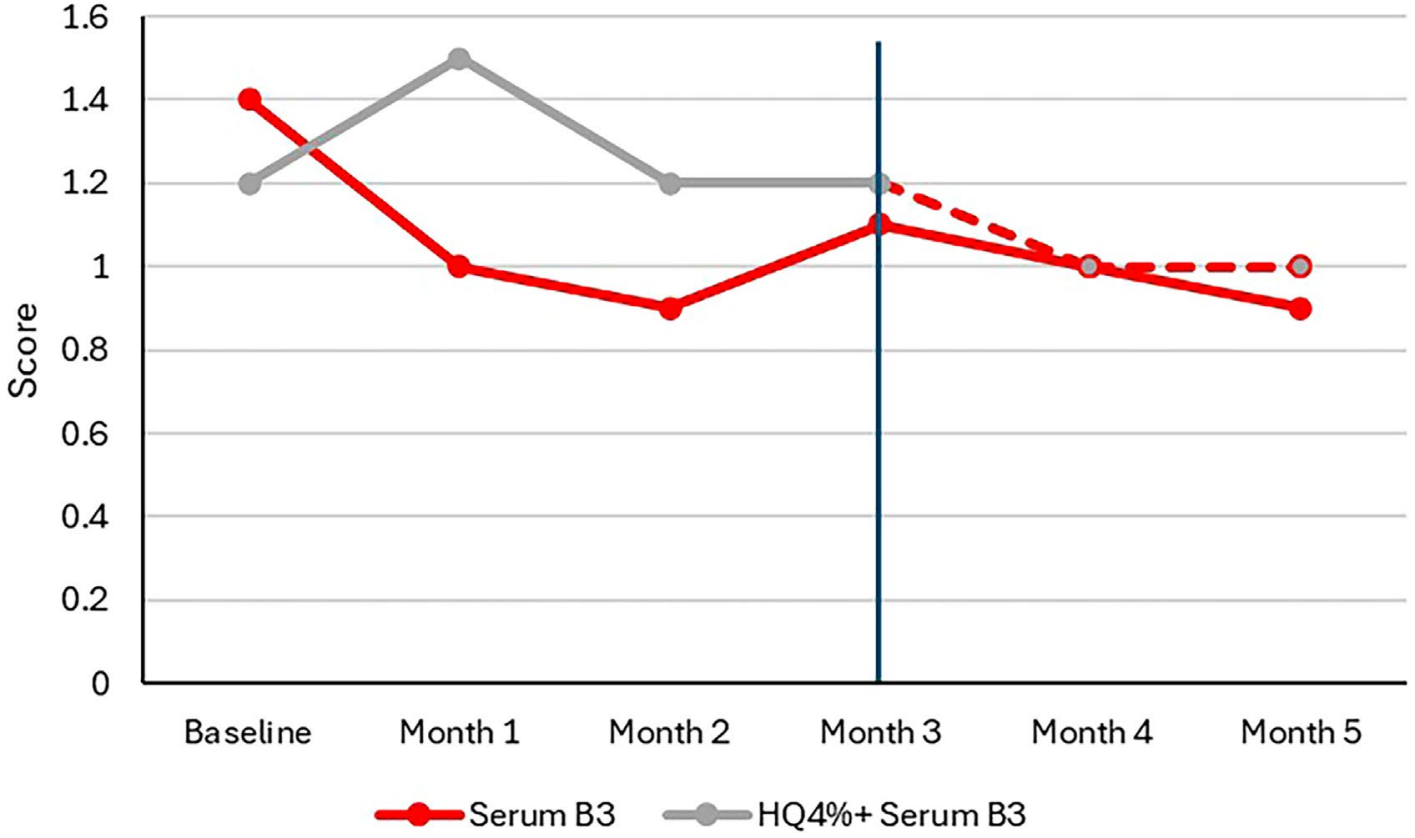
Investigator global assessment of erythema. Erythema had decreased in both groups with no significant difference after 3 months.

After 1 month of use, erythema severity decreased from 1.4 ± 1.0 at baseline to 1.0 ± 1.0 in Group 1, while it increased in Group 2 from 1.2 ± 1.0 at baseline to 1.5 ± 0.9. The between‐group difference was significant (*p* = 0.002) at that visit as well as at Month 2 (*p* = 0.027) in favor of Serum B3. At the beginning of Month 3, erythema scores were similar in both groups (Figure 2). Hyperpigmentation decreased over time in both groups, with no significant difference. When replacing HQ4% with Serum B3 in Group 2 after 3 months, a significant (*p* ≤ 0.05) greater reduction compared to Group 1 was observed at Month 4, with no significant difference between the two groups at Month 5. Global tolerance was significantly (*p* ≤ 0.05) better in Group 1 (84.8%) than in Group 2 (59.4%) during the first 3 months and was similar for both groups at Month 4 and Month 5.

**FIGURE 3:**
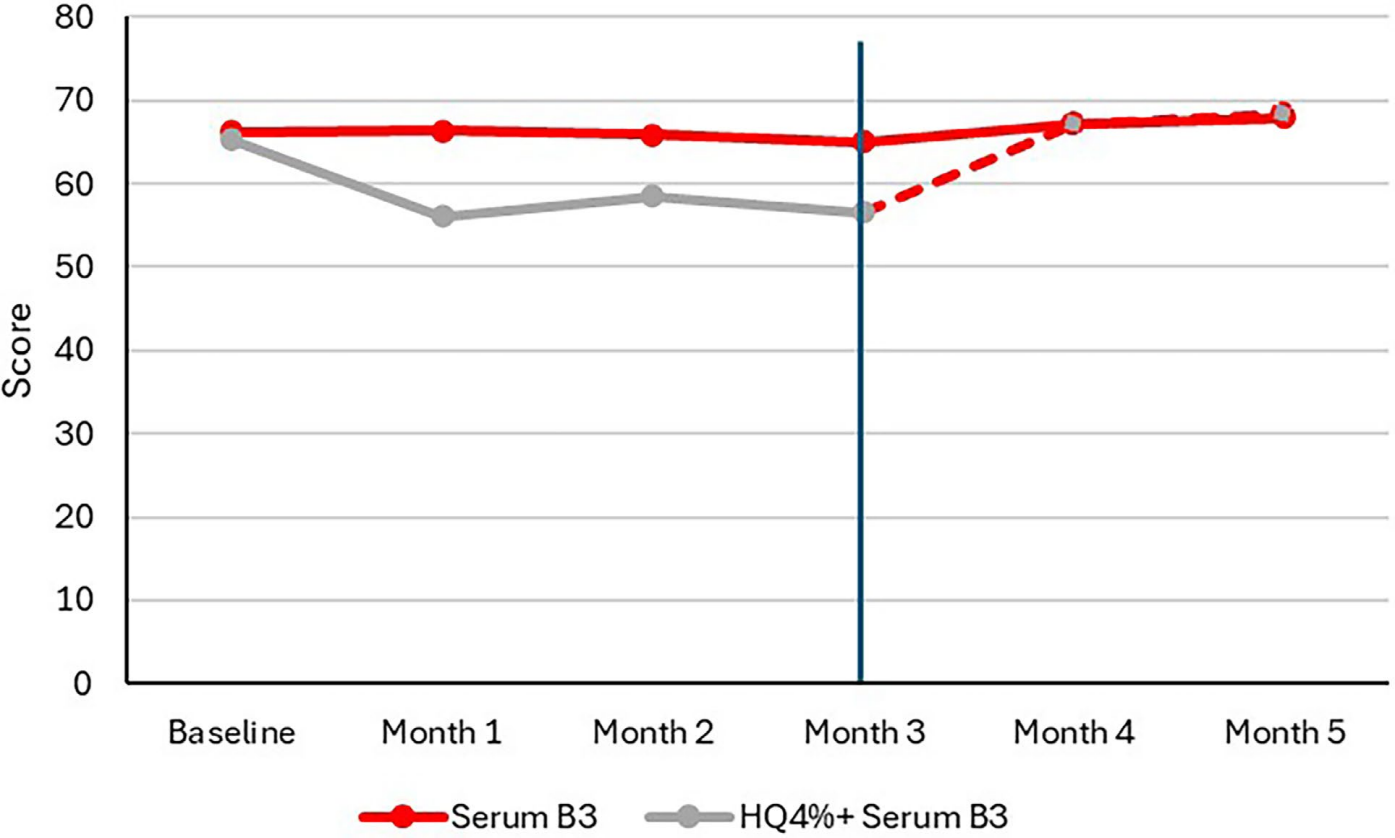
Corneometry. Skin hydration level remained unchanged with Serum B3. It significantly (*p* ≤ 0.01) decreased with HQ 4% until Month 3 and increased once Serum B3 replaced HQ 4%.

The cutaneous hydration level was maintained in Group 1 for all 5 months, while it significantly (*p* ≤ 0.01) decreased in Group 2 during treatment with HQ 4%. Hydration levels in Group 2 then increased once Serum B3 replaced HQ 4%, achieving the same levels as those measured in Group 1 at Month 4 and Month 5 (Figure 3). TEWL significantly (*p* < 0.001) improved in both groups after 3 months, with no significant between‐group difference (Group 1: 9.84 ± 2.96 g/m^2^/h; Group 2: 11.02 ± 3.47 g/m^2^/h). At Month 5, TEWL values further decreased in both groups, with no notable difference (Group 1: 8.21 ± 1.93 g/m^2^/h; Group 2: 8.67 ± 2.64 g/m^2^/h).

**FIGURE 4:**
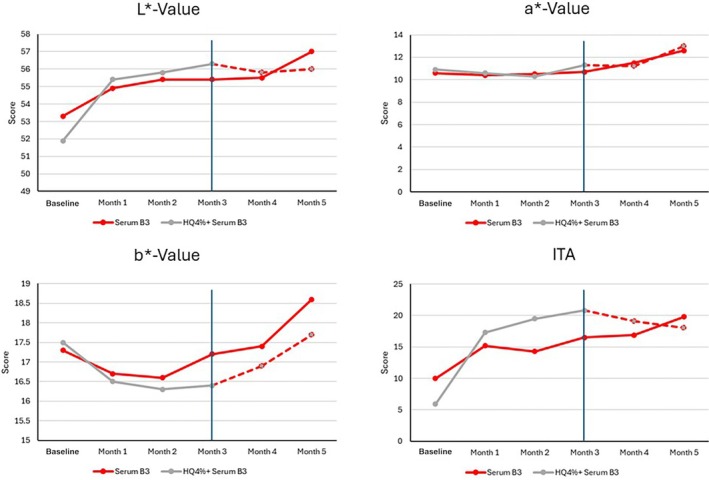
Chromametry results over time. Both Serum B3 and HQ4% provided similar chromametry results until Month 3. Serum B3 maintained the results achieved with HQ4% up to 2 months after the switch.

Colorimetric measurements demonstrated a continuous significantly higher L*, b*, and ITA° (all *p* ≤ 0.01) in Group 2 compared to Group 1 during the first 3 months, and no significant difference for a*. After 5 months, in Group 2, all chromametry values had increased, achieving similar results to those observed in Group 1. Between‐group differences were no longer statistically significant (Figure 4).

**FIGURE 5:**
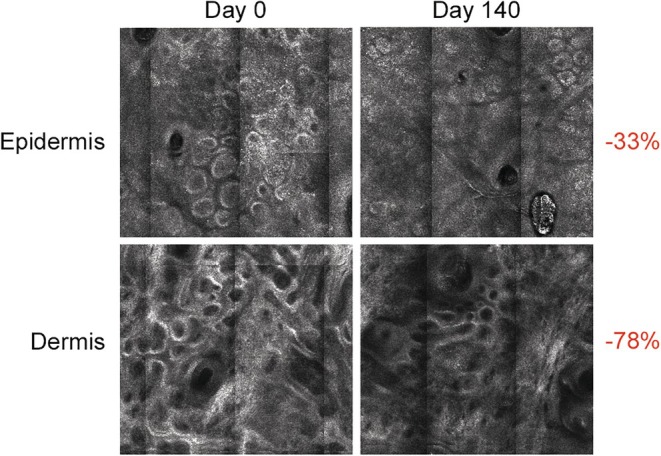
RCM images showing changes in epidermis and dermis for the serum group after 5 months of treatment; the arrow indicates the melanin halo. The melanin concentration significantly (*p* < 0.05) decreased with Serum B3 in both the epidermis and dermis.

A significant decrease (*p* < 0.05) in the melanin density was observed in both groups in the epidermis (Group1: −35.8%; Group 2: −44.3%;) and dermis (Group 1: −66.0%; Group 2: −70.9%) after 84 days of application, thereby confirming the presence of hyperpigmentation in these skin layers. In both groups, the melanin density in the epidermis (Group 1: −33.4%; Group 2: −45.9%) and dermis (Group 1: −78.6%; Group 2: −76.3%) further decreased after 140 days. Figure 5 provides an example of RCM results after 140 days of use of Serum B3.

QoL significantly (*p* < 0.001) improved in both groups, with a significant (*p* < 0.009) higher improvement in Group 2 (46.4%) than in Group 1 (23.0%) during the first 3 months, and with 84.8% (Group 1) and 81.3% (Group 2) of subjects reporting an improved QoL compared to baseline. A further QoL improvement was observed in Group 1 (35.6%) and Group 2 (37.3%) at Month 5; the between‐group difference was no longer statistically significant, and the QoL of 90.9% of subjects in Group 1 and 81.3% of subjects in Group 2 improved in comparison to baseline.

Subjects appreciated the cosmeticity of Serum B3 (96.6%) above that of HQ4% (73.3%).

**FIGURE 6:**
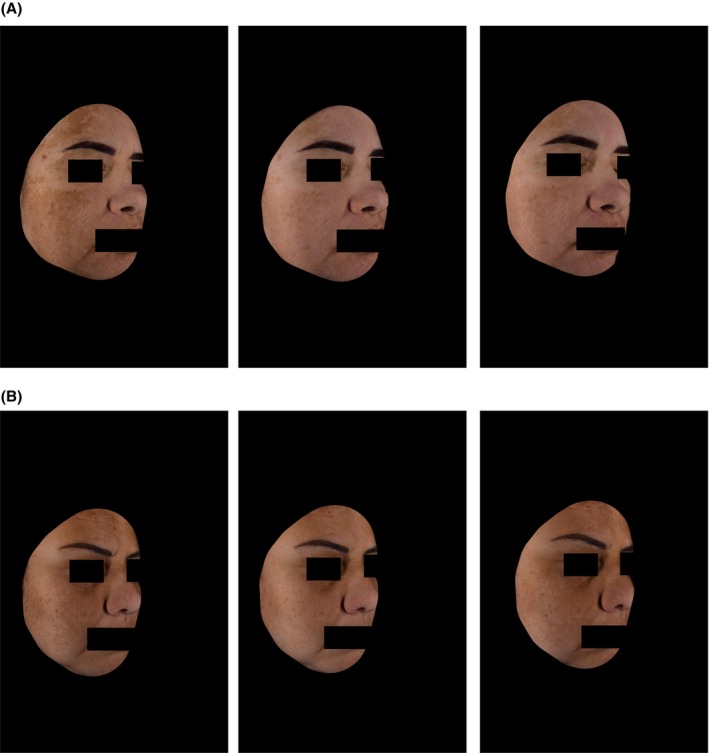
Subjects at baseline, after 3 and 5 months of treatment for Serum B3 (A) and HQ4% + B3 (B). Subjects show similar treatment outcomes after 3 and 5 months.

Figure 6 provides examples of the improvement of hyperpigmentation over time for subjects treated with Serum B3 or HQ4% + Serum B3.

## 4 Discussion

Despite the availability of various products, melasma continues to be a challenging condition to treat. Due to its chronic course, it is mandatory to have effective but also well‐tolerated products that patients can use throughout the year. Where not forbidden (i.e., European Community), HQ continues to be frequently considered the gold standard ingredient for melasma management. But its side effects, including ochronosis, erythema, scaling, and burning sensation, very often incite users to abandon long‐term treatment [12, 13, 17].

This study shows that the multicomponent Serum B3, containing 5% niacinamide, 1% TA, 0.2% vitamin CG, and 1.5% glycolic acid, provides a similar benefit in melasma as HQ4% applied for 3 months and replaced by Serum B3 for a further 2 months, as shown previously by Lima et al. for thiamidol [36]. However, skin hydration was much better with Serum B3, which was better tolerated than HQ4%, confirming the reported local tolerance issues of HQ, as reported in the literature [12, 13, 17].

The progressive decrease of the MASI and mMASI scores, the improvement of erythema and hyperpigmentation, as well as colorimetric measurements, emphasizes the depigmenting effect of both regimens during the first 3 months, and a continued improvement in both groups with no rebound once HQ4% was replaced by Serum B3 during Months 4 and 5. These results confirm that Serum B3 may present a potential beneficial alternative to HQ4% to treat melasma, as well as in the long‐term maintenance management after an initial 3‐month treatment with HQ4%.

On a dermal and epidermal level, both treatments presented similar effects, providing a significant (*p* < 0.05) pigment density reduction. This may be due to the fact that both tested products reduce melanogenesis within the epidermis and its subsequent release in the dermis, as confirmed by RCM. Despite these promising results, further studies are necessary to determine the multicomponent serum efficacy on melanin deposit better.

Subjects reported that their QoL had significantly (*p* < 0.001) improved with both products after 5 months, with significant (*p* < 0.009) between‐group differences in favor of HQ4% at Month 3, confirming the faster efficacy onset of HQ 4% in improving melasma. However, this difference was no longer significant after 5 months, thus confirming that the QoL of subjects with melasma may be strongly correlated to their skin condition. Moreover, subjects appreciated the cosmeticity of Serum B3, as reported in their self‐assessment questionnaires. Interestingly, apart from Asian skin, the study was performed on various skin types and ethnicities, with good tolerance of Serum B3 in all subgroups.

The main limitation of this study was the single‐center design as well as the absence of patients with Asian ethnicity and of male patients, which prevents drawing conclusions about these particular subgroups.

In conclusion, the present study demonstrates that Serum B3 is an effective alternative to the gold standard HQ4% in managing melasma in subjects with sensitive skin over a prolonged period. In this study, the Serum B3 regimen produced a similar depigmenting effect as a 3‐month course of topical HQ4% + 2‐month course of Serum B3 regimen. Additionally, Serum B3 significantly improved skin hydration and was better tolerated than HQ4%.

## Author Contributions

J.R. and M.S. performed the study; C.D.B. managed the study; T.P. and N.K. provided medical input; J.C.P. analyzed confocal data. All authors provided input and approved the manuscript.

## Ethics Statement

The study complied with the Principles of the Declaration of Helsinki and Good Clinical Practices. The study received local ethics committee approval (IEC Rio de Janeiro, Brazil, CEP: 22.270‐005, No.: 5.418.666, May 2022) and all subjects provided written informed consent prior to inclusion.

## Conflicts of Interest

T.P. has received honoraria and/or served as a consultant for Vichy, La Roche Posay, L'Oréal, SVR, Symrise, ACM, Caudalie, Isis Pharma, NAOS, Beiersdorf, ISDIN, Pierre Fabre and Hyphen. C.D.B. and N.K. are employees of l'Oréal Group.

## Data Availability

The data generated through this study are available upon reasonable request from Natalia Kovylkina, the corresponding author.
